# Behavior Classification and Analysis of Grazing Sheep on Pasture with Different Sward Surface Heights Using Machine Learning

**DOI:** 10.3390/ani12141744

**Published:** 2022-07-07

**Authors:** Zhongming Jin, Leifeng Guo, Hang Shu, Jingwei Qi, Yongfeng Li, Beibei Xu, Wenju Zhang, Kaiwen Wang, Wensheng Wang

**Affiliations:** 1 Agricultural Information Institute, Chinese Academy of Agriculture Sciences, Beijing 100086, China; jinzhongming@caas.cn (Z.J.); hang.shu@doct.uliege.be (H.S.); yongfeng.li@student.uliege.be (Y.L.); xuxiaobei224@163.com (B.X.); zhangwenju@caas.cn (W.Z.); kaiwen.wang@wur.nl (K.W.); 2 AgroBioChem/TERRA, Precision Livestock and Nutrition Unit, Gembloux Agro-Bio Tech, University of Liège, 5030 Gembloux, Belgium; 3 College of Animal Science, Inner Mongolia Agricultural University, Hohhot 010018, China; qijingwei_66@126.com; 4 Information Technology Group, Wageningen University and Research, 6708 PB Wageningen, The Netherlands

**Keywords:** behavior classification, grazing sheep, machine learning, sward surface heights, behavior distribution

## Abstract

**Simple Summary:**

The monitoring and analysis of sheep behavior can reflect their welfare and health, which is beneficial for grazing management. For automatic classification and the continuous monitoring of grazing sheep behavior, wearable devices based on inertial measurement unit (IMU) sensors are important. The accuracy of different machine learning algorithms was compared, and the best one was used for the continuous monitoring and behavior classification of three grazing sheep on pasture with three different sward surface heights. The results showed that the algorithm automatically monitored the behavior of grazing sheep individuals and quantified the time of each behavior.

**Abstract:**

Behavior classification and recognition of sheep are useful for monitoring their health and productivity. The automatic behavior classification of sheep by using wearable devices based on IMU sensors is becoming more prevalent, but there is little consensus on data processing and classification methods. Most classification accuracy tests are conducted on extracted behavior segments, with only a few trained models applied to continuous behavior segments classification. The aim of this study was to evaluate the performance of multiple combinations of algorithms (extreme learning machine (ELM), AdaBoost, stacking), time windows (3, 5 and 11 s) and sensor data (three-axis accelerometer (T-acc), three-axis gyroscope (T-gyr), and T-acc and T-gyr) for grazing sheep behavior classification on continuous behavior segments. The optimal combination was a stacking model at the 3 s time window using T-acc and T-gyr data, which had an accuracy of 87.8% and a Kappa value of 0.836. It was applied to the behavior classification of three grazing sheep continuously for a total of 67.5 h on pasture with three different sward surface heights (SSH). The results revealed that the three sheep had the longest walking, grazing and resting times on the short, medium and tall SHH, respectively. These findings can be used to support grazing sheep management and the evaluation of production performance.

## 1. Introduction

Sheep provide a variety of products, and their environment and health are important factors that affect production performance. As the temperature of the microenvironment increases, heat stress significantly impairs the efficiency of meat and wool production [1]. Diseases not only affect sheep, but are also a major cause of economic loss for the sheep industry [2]. The behavior of livestock can reflect their response to the environment and health [3]. Li et al. [4] pointed out that the lying time of Small Tail Han sheep increases significantly when the ambient temperature rises, so forage intake is reduced to lower heat production in sheep. When ruminal bloat happens, grazing and rumination slow down or stop, and sheep keep getting up and lying down [5]. The duration of resting behavior can reflect their social stress in animal husbandry [6]. Therefore, the continuous monitoring and analysis of sheep behavior relays their welfare and health status in a timely manner, which leads to the formulation of measures to improve their welfare, reduce economic losses and help achieve efficient and sustainable development [7].

Traditional manual observation needs an observer to record livestock behavior, which is both time- and labor-consuming and potentially has an impact on normal livestock behavior [8,9]. Moreover, it makes continuous monitoring impossible, especially when there is a large quantity and wide distribution of livestock [3]. Many studies [10,11,12,13] pointed out that computer vision, sound analysis, motion sensing, satellite positioning and other technologies have been used to improve the ability of remote, large-scope and large-scale monitoring of livestock behavior. The wearable motion sensor is more suitable for fine-scale monitoring of the behavior of grazing sheep than computer vision and satellite positioning [14], and has potential application prospects. Motion sensors have been widely used in cattle to identify behavior, and many products––for example, Lely [15], MooMonitors [16], IceTag3D™, REDI, SCR/Allflex and CowManager Sensor systems [17]—are commercially available. Due to the differences in physiology and behavior, the products of cattle monitoring cannot be applied directly to sheep [18]. Moreover, research into the behavior classification of grazing sheep is less than that of cattle [19]. As such, using motion sensors to monitor the behavior of sheep is of great interest.

The behavior monitored for sheep generally includes grazing, lying, standing, walking, ruminating, running, etc. The main factors affecting the classification accuracy of a motion sensor are the wearing position, sensor type, data collection frequency, time window size, feature construction and algorithm. However, the above factors chosen to achieve an optimum classification accuracy of sheep behavior varied in previous studies (Table 1), and very few of the trained models have been applied to continuous behavior segments in actual situations. Therefore, the aim of this study was to assess the performance of a range of machine learning (ML) algorithms (extreme learning machine (ELM), AdaBoost, stacking) in classifying walking, standing, grazing, lying and running behavior of grazing sheep at three different time windows (3, 5 and 11 s) using three different sensor data types (three-axis accelerometer (T-acc), three-axis gyroscope (T-gyr), and T-acc and T-gyr). The focus of this study was to evaluate multiple combinations of algorithms, time windows and sensor data. The optimal combination was selected to analyze the behavior distribution of three sheep grazing continuously on pasture with different sward surface heights (SSH).

## 2. Materials and Methods

### 2.1. Experimental Site, Animals, and Instrumentation

This study was approved by the Animal Ethics Committee of the University of New England, and followed the Code of Research Conduct for the University of New England, to conform to the Australian Code for Care and Use of Animals (AEC17-006).

**Figure 1:**
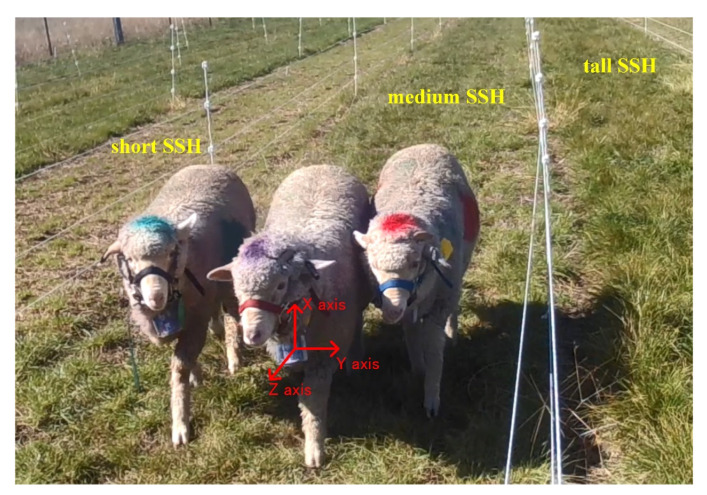
Experimental paddock on pastures with different sward surface heights (SSH). Location of inertial measurement unit (IMU) sensor and its orientation on sheep.

The experimental site was located in the ryegrass pasture (−30.5, 151.6) near the University of New England, New South Wales, Australia. To study the behavior distribution of sheep on different SSH, three paddocks of 72 m^2^ (48 m long × 1.5 m wide) were set up, and the SSH was cut to 2–3 cm (short), 5–6 cm (medium) and 8–10 cm (tall), as shown in Figure 1. A water trough was provided at one end of each paddock for the sheep to drink from.

Three 8-month-old Merino sheep of approximately 35 kg were used in this study. A designed wearable device based on InvenSense MPU-9250 was worn on the neck of sheep to collect inertial measurement unit (IMU) data. The collected data were stored in the device’s secure-digital (SD) card. MPU-9250 provides a T-acc, a T-gyr and a three-axis magnetometer. The *x*-, *y*-, and *z*-axis represent movement in vertical, horizontal, and lateral directions, respectively (as shown in Figure 1). In more detail, the collection frequency of IMU data was 20 Hz, the range of the T-acc value was ±2 g (9.8 m/s^2^), and the range of the T-gyr value was ±2000 dps (°/s). To distinguish each sheep clearly in the shooting video, blue, purple, and red livestock-marking pigments were used as markers; hence, the names B-Sheep, P-Sheep and R-Sheep were assigned. The IMU data of grazing sheep on pastures with different SSH were collected for 5 consecutive days: at the short SSH on the 15th and 16th, at the medium SSH on the 17th and 18th, and at the tall SSH on the 19 May 2017. To record the sheep behavior, a camera was fixed at one end of the paddock, while the captured videos were stored in the camera’s SD card. Sheep entered the paddock at 8:00 a.m. on each test day and the camera began to collect data for 4 h.

### 2.2. Sheep Behavior Definition and Labelling

Based on previous studies and the situation of this study, the behavior of grazing sheep was classified into five categories: walking, standing, grazing, lying and running. These behaviors are described in Table 2.

The video data of the three sheep collected on the 15th and 18th of May were labelled using the behavior labelling software developed by the authors. A camera was placed at one end of the paddock, but the sheep that were too far away or were too close together were labelled as unknown because their specific behavior could not be observed. Each video contained behavior records of the three sheep. To complete the behavior labelling of the three sheep in the video, each video had to be labelled three times. Video labelling data were linked with IMU data by the corresponding timestamp and used as the label of behavior. When constructing the dataset, all unknown labels were discarded, leaving behind only the behavior label that could be observed clearly (Table 3). Data on the running behavior in the labelled dataset were rare because the experimental site was very safe and there were no threats that would make the sheep run away. In this study, the sheep ran mainly because they touched the electrified fence of the paddock. However, this happened very infrequently, which led to a very unbalanced dataset of five behaviors.

Guo et al. [25] found that it was robust to use the model trained on a specific SSH to classify grazing and non-grazing behavior of grazing sheep on pastures with different SSH (2–10 cm). Therefore, this study adopted the data training model of three sheep on the 15th and 18th of May, and applied the trained model on the 16th, 17th and 19th of May to the 7.5 h continuous behavior segments classification of the three grazing sheep on pastures with three different SSH. To evaluate the practical application performance of the model for sheep behavior classification in continuous behavior segments, part of the data of the five behaviors were randomly labelled by video as a continuous behavior segments test dataset (CBS test dataset), which included three sheep on pasture with three SSHs walking for 1250 s, standing for 1800 s, grazing for 1800 s, lying for 1800 s, and running for 37 s.

### 2.3. Wavelet Transform Denoising

**Figure 2:**
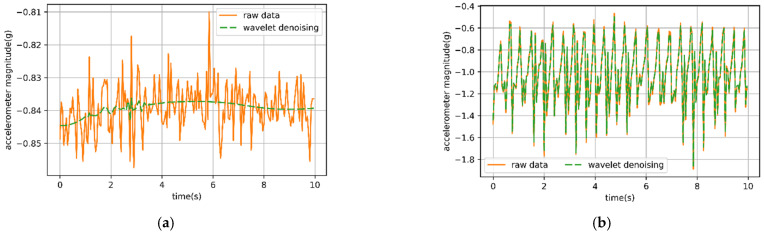
Comparison of the signal before and after wavelet denoising. (**a**) Time series of *x*-axis accelerometer signal from 20 Hz sampling rate for observed behaviors of 10 s lying; (**b**) time series of *x*-axis accelerometer signal from 20 Hz sampling rate for observed behaviors of 10 s walking.

It was necessary to reduce the noise of the collected data as the collected IMU data were inevitably disturbed by noise. The various sheep behaviors were completed by various specific movements, each represented by different T-acc and T-gyr data, so it was particularly important to save the peak signals and changing data signals for the behavior representation. Wavelet filtering effectively filtered out the noise while retaining the peak and mutation values to the maximum extent. The wavelet denoising experiment for collected IMU data was carried out using different thresholds and rules in MATLAB. Discrete wavelet db6 was selected as the basis function, and the raw data were decomposed by five layers of wavelet. According to heursure, quantization was carried out under a soft threshold [26]. In the end, wavelet reconstruction was carried out to complete wavelet transform denoising for the collected IMU data. Wavelet transformation effectively removed the high-frequency noise from the static behavioral data of sheep, while retaining change data in the dynamic behavior (Figure 2).

### 2.4. Time Window Size Selection

The sheep behavior was not instantaneous but consisted of specific movements throughout a period. During the experiment, 20 records were collected every second, but each record was obviously insufficient to represent a behavior. To solve this problem, previous studies usually used the windowing method to complete data classification, and the data in each time window were used to represent a kind of behavior. Studies found that the dynamic behavior of an animal’s daily activity changed periodically [26], so it was more reasonable to determine the time window of the specific behavior based on the movement period of the animals’ behavior. Since dynamic behavior has a stronger movement periodicity than static behavior, we analyzed the period of three dynamic behaviors (walking, running, and grazing) to determine the appropriate time window for behavior classification.

**Figure 3:**
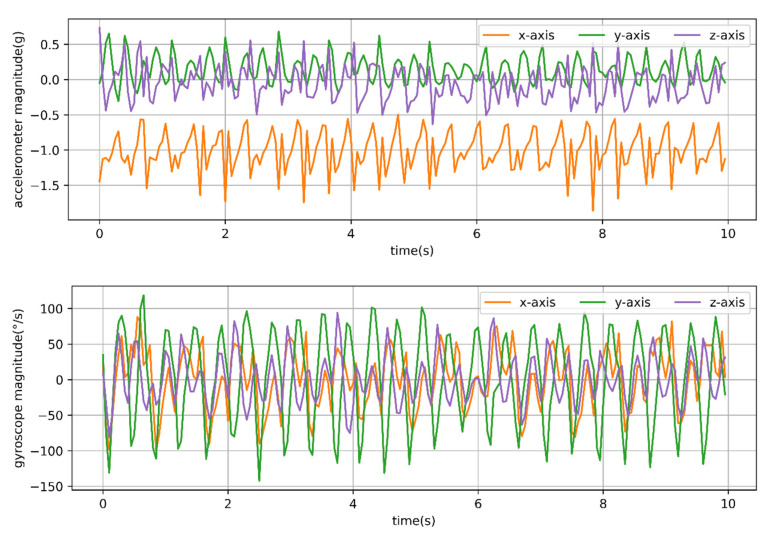
Time series of three-axis accelerometer and three-axis gyroscope signals from a 20 Hz sampling rate for observed behavior of 10 s walking. Acceleration is in g (9.8 m/s^2^) units.

It was found that the typical walking and running behaviors of sheep have strong periodicity. The T-acc and T-gyr signals of a typical 10 s of walking behavior of sheep are shown in Figure 3.

**Figure 4:**
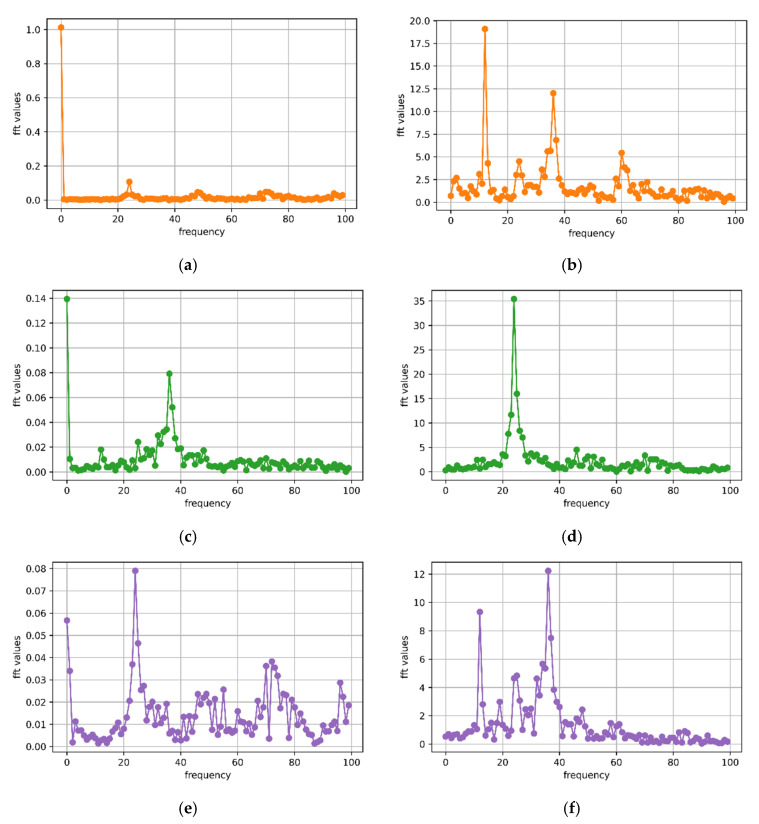
Fourier transform performed on the three-axis accelerometer and three-axis gyroscope signals of the 10 s walking behavior shown in Figure 3 to calculate the main frequency and period. (**a**) *x-*axis accelerometer: *n* = 23; *N* = 200; *f* = 2.2 Hz; *T* ≈ 0.45 s; (**b**) *x-*axis gyroscope: *n* = 12; *N* = 200; *f* = 1.1 Hz; *T* ≈ 0.91 s; (**c**) *y-*axis accelerometer: *n* = 36; *N* = 200; *f* = 3.5 Hz; *T* ≈ 0.29 s; (**d**) *y-*axis gyroscope: *n* = 24; *N* = 200; *f* = 2.3 Hz; *T* ≈ 0.43 s; (**e**) *z-*axis accelerometer: *n* = 24; *N* = 200; *f* = 2.3 Hz; *T* ≈ 0.43 s; (**f**) *z-*axis gyroscope: *n* = 36; *N* = 200; *f* = 3.5 Hz; *T* ≈ 0.29 s.

As shown in Figure 4, the *x*-, *y*- and *z*-axis signals of the T-acc and T-gyr in Figure 3 were subjected to fast Fourier transformation. At the same time, the dominant frequency and period were calculated using the Formulas (1) and (2).

(1) $$f=\left(n-1\right)\times \frac{f_{s}}{N}\left(f_{s}\;is\;the\;sampling\;frequency,\;which\;is\;20Hz\;in\;this\;study\right)$$

(2) $$T=\frac{1}{f}\;$$

**Figure 5:**
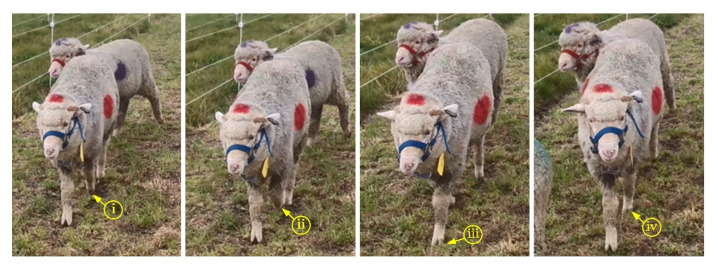
Decomposition movement of sheep’s typical walking behavior in one period. (**i**) The starting state of walking behavior; (**ii**) the sheep lifts the left leg; (**iii**) the body moves forward with the left leg; and (**iv**) the sheep lifts the right leg and moves forward with the right leg to the starting state.

The maximum and minimum periods for calculating the typical walking behavior segment (Figure 3) of sheep were 0.91 and 0.29 s, respectively. The maximum and minimum frequencies were 3.5 and 1.1 Hz, respectively. *X*-axis accelerometer (*x*-acc), *y*-axis gyroscope (*y*-gyr) and *z*-axis accelerometer (*z*-acc) signals had similar periods, while *y*-axis accelerometer (*y*-acc) and *z*-axis gyroscope (*z*-gyr) signals had the same periods. Observing the walking behavior of sheep through the video found that (i) to (iv) in Figure 5 was considered a complete period of a sheep’s walking. It was considered that the walking time was about 0.29 s from (i) to (ii), about 0.45 s from (i) to (iii), and about 0.91 s from (i) to (iv) in the 10 s walking behavior segment shown in Figure 3. A total of 24 typical walking segments of sheep were observed by video, and the average period was 0.93 s and the maximum period was 1.25 s.

**Figure 6:**
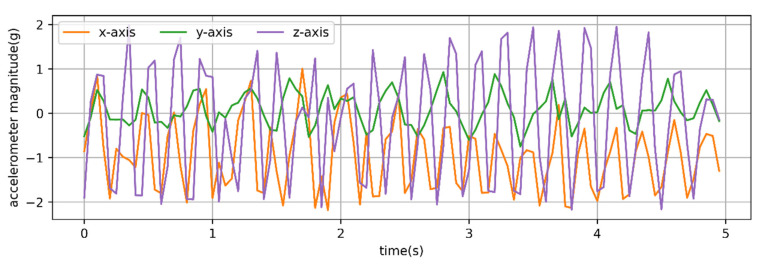

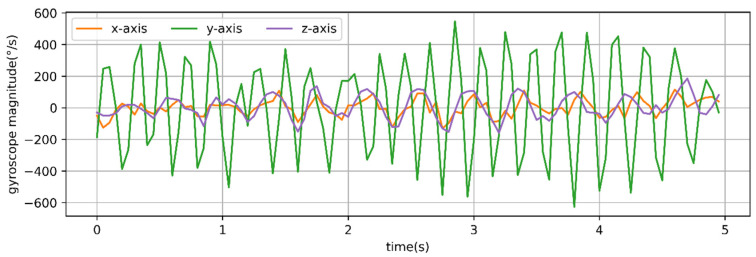
Time series of three-axis accelerometer and three-axis gyroscope signals from a 20 Hz sampling rate for observed behavior of 5 s of running. Acceleration was in g (9.8 m/s^2^) units.

**Figure 7:**
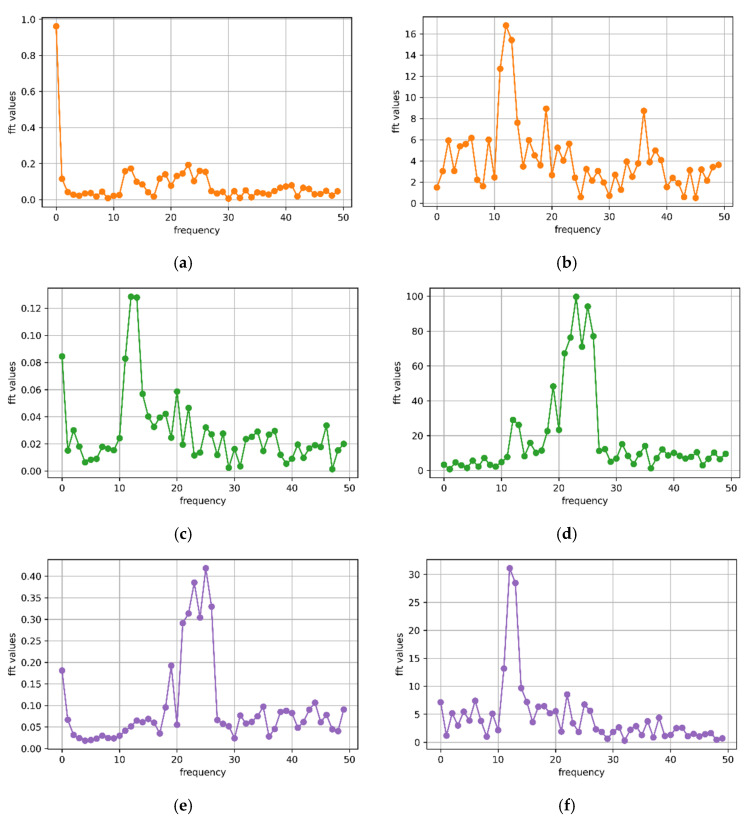
Fourier transform analysis of three-axis accelerometer and three-axis gyroscope signals for 5 s of running behavior, as shown in Figure 6, calculating the main frequency and period. (**a**) *x*-axis accelerometer: *n* = 23; *N* = 100; *f* = 4.4 Hz; *T* ≈ 0.23 s; (**b**) *x*-axis gyroscope: *n* = 12; *N* = 100; *f* = 2.2 Hz; *T* ≈ 0.45 s; (**c**) *y*-axis accelerometer: *n* = 12; *N* = 100; *f* = 2.2 Hz; *T* ≈ 0.45 s; (**d**) *y*-axis gyroscope: *n* = 23; *N* = 100; *f* = 4.4 Hz; *T* ≈ 0.23 s; (**e**) *z*-axis accelerometer: *n* = 25; *N* = 100; *f* = 4.8 Hz; *T* ≈ 0.21 s; (**f**) *z*-axis gyroscope: *n* = 12; *N* = 100; *f* = 2.2 Hz; *T* ≈ 0.45 s.

The T-acc and T-gyr signals of a typical 5 s of running behavior of sheep are shown in Figure 6. The *x*-, *y*- and *z*-axis signals of T-acc and T-gyr were, respectively, subjected to fast Fourier transformation, as shown in Figure 7. At the same time, the dominant frequency and period were calculated.

The *x*-acc, *y*-gyr and *z*-acc signals that were used to calculate the typical running behavior segment of sheep in Figure 6 had similar periods, while the *x*-axis gyroscope (*x*-gyr), *y*-acc and *z*-gyr signals had the same periods. The maximum and minimum periods were 0.45 and 0.21 s, respectively. The maximum and minimum frequencies were 4.8 and 2.3 Hz, respectively. The maximum period of the running behavior segment of the sheep (Figure 6) was about half of that of the walking behavior segment (Figure 3).

**Figure 8:**
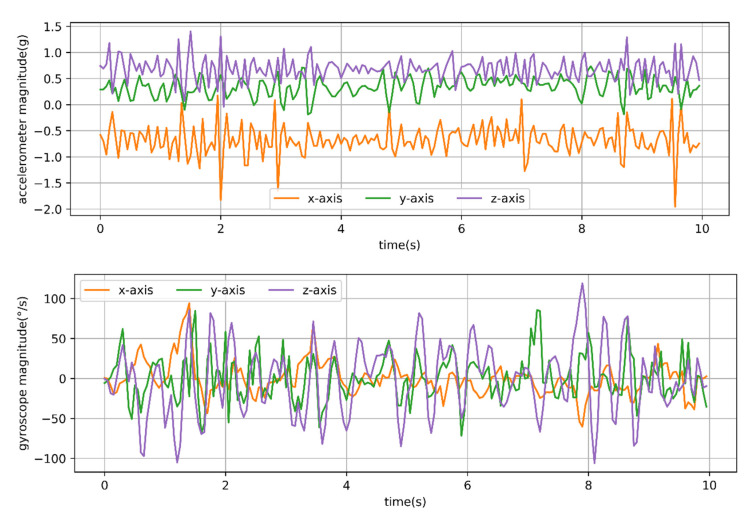
Time series of three-axis accelerometer and three-axis gyroscope signals from a 20 Hz sampling rate for observed behavior of 10 s of grazing. Acceleration was in g (9.8 m/s^2^) units.

Figure 8 presents the T-acc and T-gyr signals of a typical 10 s of grazing behavior of sheep. The *x*-, *y*- and *z*-axis signals were, respectively, subjected to fast Fourier transformation, and the dominant frequency and period were calculated at the same time.

Compared with typical walking and running behavior, grazing had no significant periodicity. Moreover, through observation, it was found that the period of sheep grazing behavior on pasture with different SSH was also different. Therefore, the period of sheep grazing behavior was mainly determined by video observation. The process of observing a sheep’s grazing behavior could be roughly divided into: (i) biting once or several times and then swallowing; (ii) biting once or several times, then chewing and finally swallowing; (iii) grazing, then biting once or several times and finally swallowing; (iv) grazing, then biting once or several times and then chewing (or chewing while foraging), and finally swallowing. Due to the biting movement of sheep, the video was easily observed, and the time interval between two biting movements was taken to be the grazing period. A total of 41 grazing segments of sheep in the video were observed, and the duration of each one divided by the number of biting movements was taken as the period of grazing behavior: the maximum period was 2.15 s, which was longer than the maximum period of walking by 1.25 s. Considering that the period of walking behavior was about twice that for running behavior, the maximum period for observing dynamic behavior was 2.15 s. Therefore, a minimum time window of 3 s was enough to satisfy a behavioral movement period; time windows of 3, 5 and 11 s were used for time window comparison, being the maximum period of 2.15 s rounded up 1, 2, and 5 times.

### 2.5. Classification Feature Construction

Based on the labelled T-acc data and T-gyr data, the feature datasets were constructed out of the time and frequency domains with time windows of 3, 5 and 11 s. Table 4 shows the selected time and frequency domain features.

A total of 63 features were constructed based on the T-acc data, 48 features were constructed based on the T-gyr data, and 111 features were constructed based on the T-acc and T-gyr data. In order to compare the accuracy of three kinds of sensor data for behavior classification, AdaBoost was used to rank the feature importance of behavior classification in T-acc data, and T-acc and T-gyr data. The top 48 important features were selected to construct its feature dataset.

**Figure 9:**
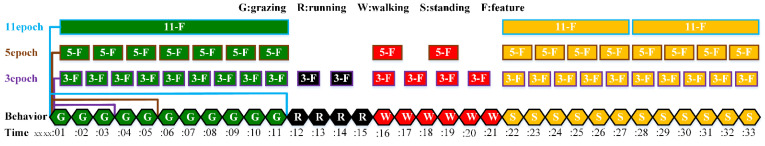
The features calculation process of three time windows (3, 5 and 11 s) on the 33 s observed behavior segments.

Nine behavioral feature datasets were constructed for three different time windows and three different kinds of sensor data combinations: in the constructing the datasets, the larger the time window, the fewer the rows of feature data (Figure 9). There were 50,302 rows of feature data with a 3 s time window; 46,101 rows of feature data with a 5 s time window; and 23,015 rows of feature data with an 11 s time window in the labelled behavioral data on the 15th and 18th of May. The duration of the running segments did not exceed 11 s, and only a few of them exceeded 5 s. As a result, this sheep behavior could not be constructed within an 11 s time window in the labelled data, and only a few running behavior features could be constructed within a 5 s time window. Therefore, it was not included in the feature datasets with an 11 s or 5 s time window. The nine behavioral feature datasets were standardized: 80% of each was used as the training and 20% as the test dataset.

### 2.6. ML Classification Algorithms

Ensemble learning is a technique for improving prediction performance by constructing and integrating multiple machine learners [27]. According to different integration strategies for different machine learners, ensemble learning can be divided into boosting [28], bagging [29] and stacking [30]. Boosting and bagging usually integrate homogeneous learners: boosting adopts sequence integration, and bagging adopts parallel integration. Stacking integrated heterogeneous learners is a hierarchical structure, and the outputs of multiple heterogeneous learners in the first layer are used as learner inputs in the second layer of the training model. The integration of multiple learners can reduce the possible deviation of a single classifier when dealing with unbalanced data and prevent over-fitting, resulting in a better performance than a single algorithm. Therefore, more studies apply ensemble learning to the classification learning of unbalanced data [31,32].

The ELM method has a ML training speed thousands of times faster than that of a traditional back-propagation neural network. It is based on a generalized, single-hidden layer, feedforward neural network and has good generalization performance [33,34,35].

ELM, AdaBoost (a concrete implementation of the Boosting algorithm) [36] and stacking were used to classify sheep behavior in this study. The basic learner of the stacking algorithm adopted AdaBoost, random forest (RF, an improvement on bagging), and support vector machine (SVM), which has been applied well in previous research on sheep behavior classification. The secondary learner of the stacking algorithm adopted ELM. Trained ELM, AdaBoost, and stacking were compared for accuracy and practical application in sheep behavior classification.

### 2.7. Performance of the Classification

The accuracy of the trained models was evaluated on the test dataset and the CBS test dataset, and the evaluation indexes were accuracy and Kappa value, which is an index used to test whether the model prediction is consistent with the actual values. Accuracy was calculated using Formula (3):

(3) $$\mathrm{accuracy}=\frac{\mathrm{TP}+\mathrm{TN}}{\mathrm{TP}+\mathrm{FP}+\mathrm{FN}+\mathrm{TN}}$$

True positive (TP) indicated that both the actual category and the model prediction category were positive. True negative (TN) indicated that both were negative. False positive (FP) indicated a positive model prediction category, but a negative actual category. False negative (FN) indicated a negative model prediction category, but a positive actual category. The Kappa value was calculated based on a confusion matrix, and the calculated result was between −1 and 1, but usually between 0 and 1. The larger the value, the higher the model classification accuracy. The Kappa value is very suitable for evaluating the performance of the model for classifying the unbalanced quantity of samples in various categories [37].

The classification performance of each behavior was evaluated for precision, recall and F-score, and calculated by using Formulas (4)–(6).

(4) $$\mathrm{Precision}=\frac{\mathrm{TP}}{\mathrm{TP}+\mathrm{FP}}\;$$

(5) $$\mathrm{Recall}=\frac{\mathrm{TP}}{\mathrm{TP}+\mathrm{FN}}\;$$

(6) $$F-\mathrm{score}=2\times \frac{\mathrm{Precision}\times \mathrm{Recall}}{\mathrm{Precision}+\mathrm{Recall}}$$

## 3. Results

### 3.1. Model Training and Test Results

The training dataset was used to train the model, during which a 5-fold cross validation was conducted to select the optimal hyperparameters; the test dataset was used to evaluate the performance of the trained model. The performance of the trained models on the test dataset is shown in Table 5.

It was found that the accuracy of the three models in the three time windows from three types of sensor data were all above 90%, and the Kappa values were above 0.85. The larger the time window, the higher the classification accuracy. Data classification accuracy using T-acc and T-gyr sensors was higher than using them separately, and accuracy using T-acc was higher than using T-gyr. Model accuracy was stacking > AdaBoost > ELM.

### 3.2. Practical Application of the Trained Models

**Figure 10:**
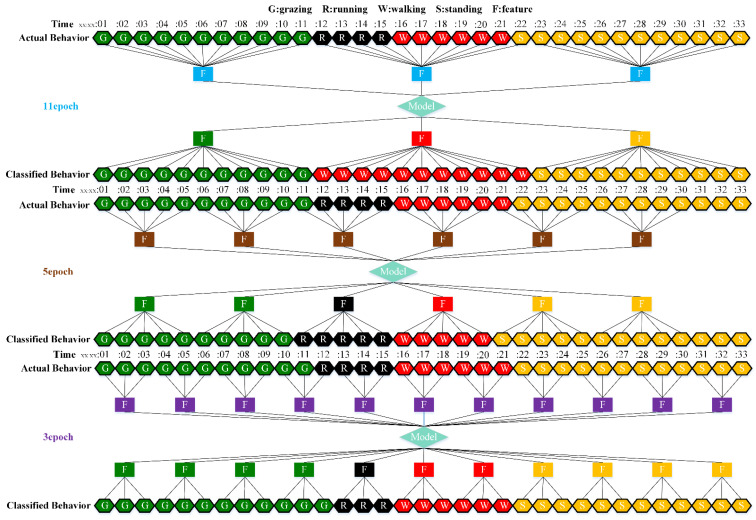
The process of 33 s of continuous behavior segment classification using 3, 5 and 11 s time windows with jump-moving. Assuming that the model accurately classified each behavioral feature, the classification accuracy at 3, 5 and 11 s was 97.0, 93.9 and 84.8%, respectively.

**Figure 11:**
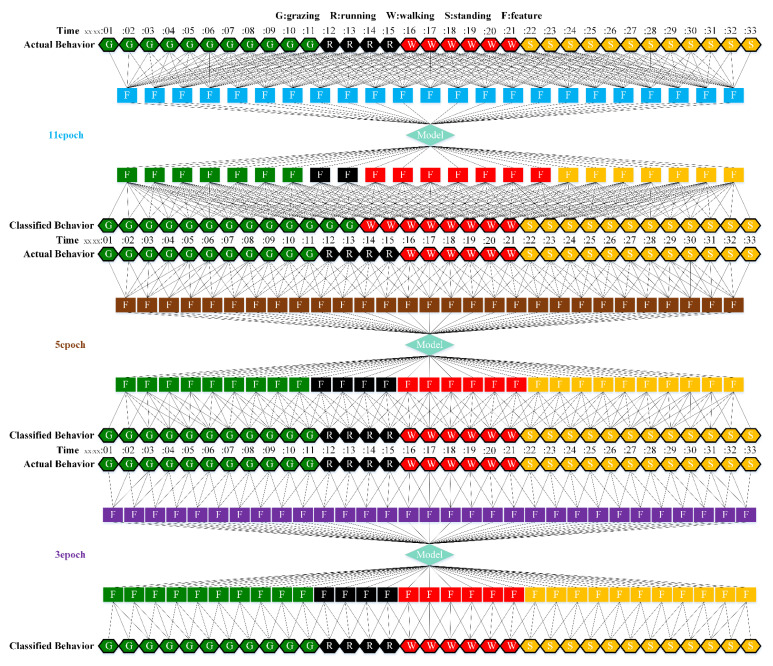
The process of 33 s of continuous behavior segment classification using 3, 5 and 11 s time windows with slide-moving. Assuming that the model accurately classified each behavioral feature, the classification accuracy at 3, 5 and 11 s was 100, 100 and 87.9%, respectively.

The 27 trained models were applied to the behavior classification of three grazing sheep on pasture with three different SSH from 9:00 a.m. to 4:30 p.m. on the 16th, 17th and 19th of May. The moving mode of the time window during classification comprised jumping and sliding. For example, the sheep touched the electrified fence during grazing, then ran for 4 s, walked for 6 s, and finally stood up. Assuming that the model classified every behavior feature with 100% accuracy, behavior classification with 3, 5 and 11 s time window jump-moving is shown in Figure 10, and classification with slide-moving is shown in Figure 11.

Theoretically, the slide-moving window classification accuracy should be higher, so the slide-moving window was used when using the 27 trained models for continuous behavior segments classification. The final classification result at each moment was determined by the behavior with the highest prediction score among all behaviors classified by the trained model for all time windows containing that moment, as shown in Figure 11. The results of 27 trained models on the CBS test dataset are shown in Table 6.

When applying the trained models to continuous behavior segments, the accuracy on the CBS test dataset obviously decreased. Based on T-acc and T-gyr data, the 3 s time window and stacking model had the highest sheep behavior classification accuracy of 87.8% and a Kappa value of 0.836. The larger the time window, the lower the classification accuracy, which was contrary to the results in the test dataset. The accuracy of classification by using T-acc data was higher than using T-gyr data, which was the same as the results in the test dataset. In most instances, the classification accuracy of using the two kinds of sensor data was higher than that by using them separately. Stacking and ELM models performed better on the CBS test dataset.

The classification accuracy (Table 7) of the optimal model for each behavior across the three time windows was calculated, and the main reason for the decline in performance was the confusion of standing and lying behavior. Although the training samples of running behavior were very few, the F-score of classification still reached 82.4% in practical application due to its very special features.

The accuracy of the trained models on the CBS test dataset drops obviously compared to the classification accuracy on the test set. The models that performed the best classification at the 3, 5 and 11 s time windows were tested to see if they were over-fitted because of having too many features. Using the top 12 important features from T-acc and T-gyr data to retrain these models and test the accuracy of the trained models on the test dataset and the CBS test dataset, the results are presented in Table 8. It was found that when the number of features was reduced from 48 to 12, the performance of the trained model on the test dataset was not significantly affected, but the accuracy dropped obviously on the CBS test dataset. Compared with 12 features, training the model with 48 improved the practical performance of sheep behavior classification in continuous behavior segments.

### 3.3. Behavior Classification of Three Grazing Sheep on Pasture with Three Different SSH

**Figure 12:**
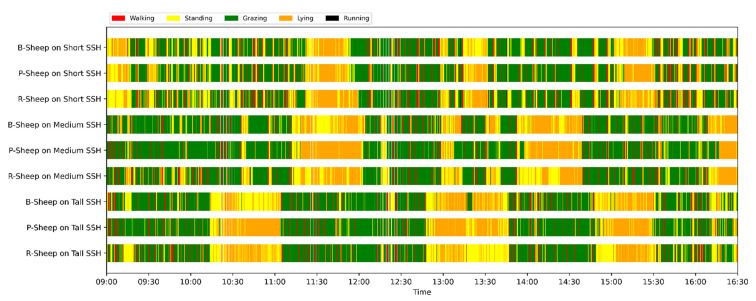
Behavior distribution of three grazing sheep on pastures with three different sward surface heights (SSH) classified by the trained stacking model.

We selected the combination (trained stacking model, 3 s time window, T-acc and T-gyr data) with the best classification performance on the CTB test dataset to classify the behavior of three grazing sheep for 7.5 h (from 9:00 a.m. to 4:30 p.m.) each on pasture with three different SSH. The classification results are shown in Figure 12.

The behavioral distribution of the three sheep on pasture with same SSH was similar, but grazing behavior on short SSH was relatively scattered with a short duration for each bout, and more continuous long-term grazing was found on medium and tall SSH. Walking behavior was usually mixed with grazing behavior. Sheep would stand or lie down after grazing for a period of time, accompanied by rumination, and then start grazing repeatedly.

To quantitatively analyze the behavior classification of the three sheep on pasture with three different SSH, the average time each sheep spent grazing in 7.5 h was counted. Because of the misclassification between standing and lying behaviors, the standing and lying behaviors were combined into resting behavior. As shown in Table 9, the behavioral distribution of the three sheep had some common characteristics: the longest walking time was on short SSH, the longest grazing time was on medium SSH, and the longest resting time was on tall SSH. Individual differences were found in which the R-Sheep had the shortest grazing and the longest walking times, while the P-sheep had the longest grazing and the shortest resting times.

## 4. Discussion

### 4.1. Time Window Size and Sensor Type

In this study, several kinds of time windows, sensor data and ML algorithms were used to classify five behaviors of grazing sheep. Depending on the maximum period of the dynamic behavior, we used 3, 5 and 11 s time windows. The results demonstrated that the larger the time window, the higher the behavior classification accuracy on the test dataset. This was consistent with the related studies. For example, Fogarty et al. [3] compared the 5 and 10 s time window and found that the 10 s one classified grazing, lying, standing and walking behaviors with higher accuracy. Walton et al. [22] compared the time windows of 3, 5 and 7 s, and found that the 7 s time window was more accurate for classifying walking, standing and lying behavior. This was because single-behavior windows [38] were usually used during model training and testing, and the behavioral features of the large window were more typical, which could increase the discrimination between each time window segment. However, the large time window resulted in less data available for training and validation because the larger the time window, the more it spanned several behaviors, and it was usually removed from the dataset (Figure 9). The time window usually affected the accuracy of behavioral classification depending on the frequency of data collection [22]. According to the different data collection frequencies and time windows used to achieve the best classification performance in previous studies, it was found that the rows of raw data contained in each time window were usually more than 100 [10,18].

When the trained models were applied to the CBS test dataset, the smaller the time window, the higher the accuracy (3 > 5 > 11 s). This was completely opposite to the performance of the trained models on the test dataset, which was a new finding of this study. The duration of each continuous behavior in the actual grazing process could not all be greater than or equal to 11 s, which led to the inclusion of two or more behaviors in a single 11 s time window [39]. This inevitably resulted in the misclassification of behaviors with durations less than 11 s (as shown in Figure 11), degrading the classification performance of trained models on the CBS test dataset. Therefore, when classifying sheep behaviors by IMU sensors, attention should be paid to the balance between the time window with the highest model training accuracy and the shortest duration of each behavior. To precisely classify the sheep behavior at every moment, the time window should be long enough to accommodate the maximum movement period of each behavior, but not too large, because the smaller the time window, the more sensitive it is to behavior classifications, especially for grazing sheep that have frequent movement changes. If the time windows were too large, some short-duration behaviors could not be precisely classified. We should pay more attention to the behavior classification performance in the CBS test set, which is more in line with the actual application and more conducive to commercialization [10].

We compared three kinds of sensor data (T-acc, T-gyr, T-acc and T-gyr) for sheep behavioral performance in this study. The result showed that the highest accuracy using T-acc, T-gyr, T-acc and T-gyr data was 84.8, 82.8 and 87.8%, respectively. The accuracy of sheep behavior classification using T-acc data was higher than that for T-gyr data. Many studies have used T-acc data for sheep behavior classification with good accuracy [8,9,10,20]. Using two types of sensor data at the same time was beneficial for improving classification accuracy in most cases. Similarly, Mansbridge et al. [18] and Walton et al. [22] have argued that using both a gyroscope and accelerometer should improve the classification accuracy of some behaviors, such as lying and eating. Nevertheless, some studies have reported that gyroscopes increase power consumption and sometimes do not deliver major classification performance improvements [40,41]. This indicates that we need to determine the type of sensor to use based on the specific behavior we want to classify.

### 4.2. Sheep Behavior Classification Algorithm

**Figure 13:**
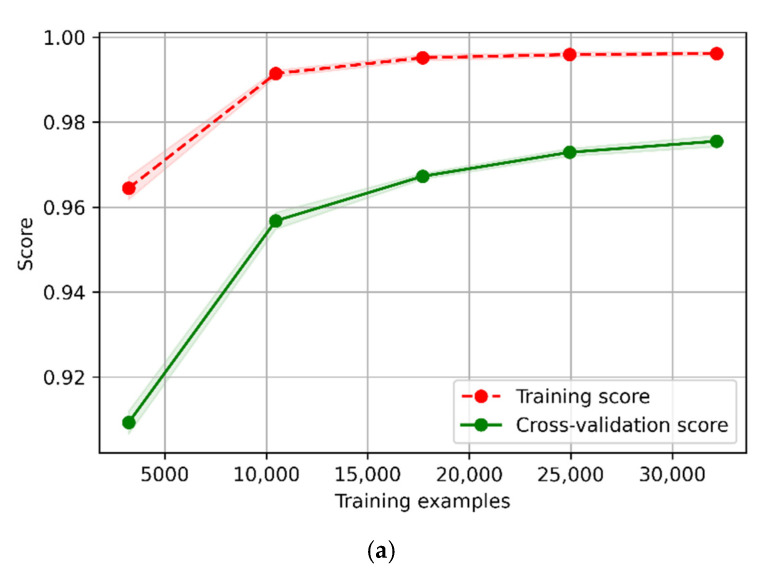

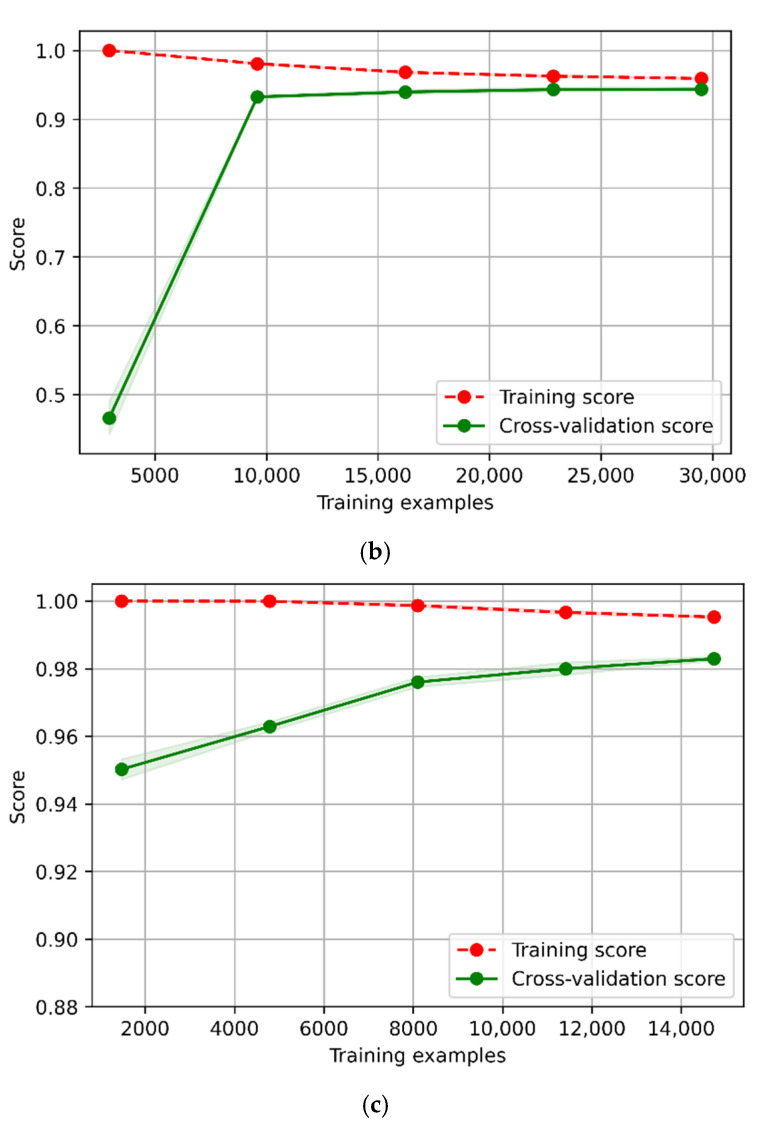
Learning curves for 5-fold cross-validation of three models (with the best performance on three different time windows) on the three-axis acceleration and gyroscope data training set. (**a**) Learning curve of stacking by 5-fold cross-validation on 3 s time window; (**b**) learning curve of ELM by 5-fold cross-validation on 5 s time window; (**c**) learning curve of ELM by 5-fold cross-validation on 11 s time window.

**Figure 14:**
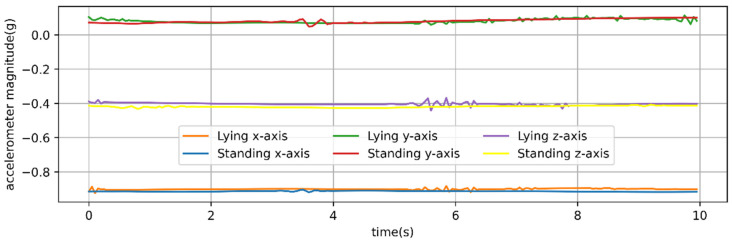
Time series of three-axis accelerometer signals from a 20 Hz sampling rate for 10 s of observed behaviors of B-Sheep standing and P-Sheep lying. Acceleration was in g (9.8 m/s^2^) units.

The results from this study showed that, compared with the classification accuracy in the test dataset, the accuracy of the trained models on the CBS test dataset decreased obviously, but not because the trained models were over-fitted. In regards to selecting model hyperparameters by five-fold cross-validation on the training data dataset, the learning curves of optimum hyperparameters of a stacking model 3 s time window and an ELM model 5 s and 11 s time window are shown in Figure 13. The accuracy of the trained models on the test dataset was similar to that on the validation dataset and had not decreased, which indicated that the trained models were not over-fitted. In the practical application, the classification accuracy of the trained models on the CBS test dataset decreased, which was mainly caused by the confusion of standing and lying behaviors as the movement of standing and lying had very similar neck movements (Figure 14). Even though the same three sheep were used during the entire experiment, the device they wore had to be taken off every day to output data and put on again the next day, which led to different wearing positions for each sheep every day. The device position has a significant influence on standing and lying behaviors, which are usually difficult to classify with ear tags and collar-mounted sensors [3,42]. As can be seen in Table 7, as the time window became larger, the precision of lying behavior increased from 75.8 to 92.5%, while the recall decreased from 85.4 to 52.6%, and the precision of standing behavior decreased from 81.9 to 63.3%, indicating that more lying behaviors were misclassified as standing behaviors. The F-score of standing behavior decreased from 77.1 to 75.9%, and the F-score of lying behavior decreased from 80.3 to 67.1%. This contrasts with Walton et al. [22], where the recall of standing and lying increased as the time window became larger, and the F-score range of standing and lying was between 94% and 97%. At the same time, it was also found that the recall of walking behavior decreased obviously in the 11 s time window, and the precision of grazing behavior also decreased, indicating that some walking behavior was misclassified as grazing behavior. This was because the misclassification mainly existed between dynamic and static behaviors. The classification performance of grazing was the best of the five behaviors, which was consistent with the findings of Fogarty et al. [3]. In order to achieve robust and high-performing classification models, more balanced data for each behavior from more sheep need to be collected to train and validate the behavior classification models [8,18].

As for the three classification algorithms, the accuracy of stacking and AdaBoost on the test dataset were high, as was the classification accuracy of stacking and ELM on the CBS test dataset, indicating that stacking and ELM showed better robustness for sheep behavior classification. These results provided a new reference for the algorithm selection of sheep behavior classification since these two algorithms were hardly reported in previous studies on sheep behavior classification [10].

### 4.3. Behavior Classification of Grazing Sheep on Pasture with Different SSH

The three merino sheep had a walking behavior while grazing (as shown in Figure 12). By observing the video, it was found that they avoided grazing facing the sun. They always grazed along the long side of the paddock with their backs to the sun, and returned when they reached the short side of the paddock. When they returned, they did not graze much since they now faced the sun. They walked for a distance to the other short side and then turned around to graze continually with their backs facing the sun. It may be the case that they were changing the incident direction and area of solar radiation by adjusting their posture, which is an effective way for animals to adjust the amount of environmental radiant heat to maintain a constant body temperature [4,43].

Compared with medium and tall SSH, the grazing behavior on the short SSH was relatively dispersed and continuous grazing time was shorter, possibly because insufficient grass forced them to search for new grass more frequently, which resulted in the highest proportion of walking behavior time. Animut et al. [44] have reported that decreasing herbage allowance [45] increases the number of sheep’s steps. Moreover, the grazing time on short SSH was less than on medium SSH, indicating that sheep might not eat enough grass on short SSH. This was supported by the increase in running on short SSH, as a result of trying to eat the grass outside the paddock and being electrocuted. The sheep ran mainly because they touched the electrified paddock. The grazing time for sheep on tall SSH was less than that on the medium SSH, but the resting time was the longest, indicating that sheep took less time to consume enough grass. This was also the conclusion of Wang et al. [46], who found that the relationship between grazing time and SSH (13 ≥ SSH ≥ 5 cm) was parabolic, opening upward. However, why the three sheep in this study had similar average grazing times on both tall and short SSH requires further investigation. Since only three sheep were used for analysis, statistical tests could not be performed. To overcome this limitation, behavioral data collected from more individual sheep are expected in future studies.

It was found that under the same sward conditions, the forage intake of grazing livestock correlated positively with grazing time and speed, and individual forage intake [47,48]. Given that the three sheep were similar in age and weight, and assuming that the individual grazing speed and grass intake were the same, the grass intake of three sheep could be inferred from the predicted grazing time of sheep: P-Sheep > B-Sheep > R-Sheep. R-Sheep always had the shortest grazing and longest walking times, which prompted us to study the reasons for this phenomenon further. Our results demonstrated a good potential for detecting individual differences in behaviors, and will facilitate the monitoring of grazing sheep health, support farm decision-making and improve production efficiency.

## 5. Conclusions

In the training process of the sheep behavior classification model, testing the trained model on continuous behavior segments was very important for evaluating the generalization ability and practical application performance of the trained model. Sensor type, time window size, time window moving mode and algorithms all affected the accuracy of continuous behavior segments classification. The accuracy of behavior classification using T-acc data was higher than that for T-gyr data, and still higher when both data were used simultaneously. The time window should be larger than the movement period of the behavior. The 3 s time window showed higher accuracy than the 5 or 11 s time windows when classifying the behavior of each second in continuous time. Stacking and ELM showed stronger robustness on the CBS test dataset. The approach followed in this study can be used to study individual behavior of sheep. In follow-up research, it will be necessary to collect more data on individual sheep, to optimize the unbalance of training data datasets, and to explore the method of judging the health of sheep through behavior time.

## Figures and Tables

**Table 1:** Overview of algorithms and other parameters for sheep behavior classification to achieve the best classification accuracy.

Sensor Position	Sensor	Data Collection Frequency	Time Window	The Number of Features Finally Used for Classification	Type of Behavior	Algorithm	Accuracy	Source
Neck	Tri-axial accelerometer	100 Hz	5.12 s	10	Lying Standing Walking Running Grazing	Quadratic Discriminant Analysis	89.7%	Marais et al. [20]
Under-jaw	Three-axis accelerometer	5, 10, 25 Hz	5 s	5	Grazing Lying Running Standing Walking	Decision Tree	85.5%	Alvarenga et al. [8]
Neck	Three-dimensional accelerometer	100 Hz	5.3 s	27	Standing Walking Grazing Running Lying	Linear Discriminant Analysis	82.40%	Le Roux et al. [21]
Ear Neck	Tri-axial accelerometer	32 Hz	5, 7 s	44	Walking Standing Lying	Random Forest	95%F-score: 91–97%	Walton et al. [22]
Neck	Tri-axial accelerometer and gyroscope	16 Hz	7 s	39	Grazing Ruminating	Random Forest	92%	Mansbridge et al. [18]
Ear Neck Leg	Tri-axial accelerometer	12 Hz	10 s	14	Grazing Standing Walking	Quadratic Discriminant Analysis	94–99%	Barwick et al. [9]
Under-jaw	Three-axial accelerometer and a force sensor	62.5 Hz	30 s	15	Grazing Ruminating Other activities	Discriminant Analysis	89.7%	Decandia et al. [19]
Rear	Accelerometers	40 Hz	3 s	30	Foraging Walking Running Standing Lying Urinating	Random Forest	0.945(Kappa value)	Lush et al. [23]
Ear	Accelerometers	12.5 Hz	10 s	19	Grazing Lying Standing Walking	Support Vector Machine	76.9%	Fogarty et al. [3]
Neck	Three-axial accelerometer and ultrasound transducer	50 Hz	5 s	11	Infracting Eating Moving Running Standing Invalid	Decision Tree	91.78%	Nóbrega et al. [24]

**Table 2:** Definition of the five behaviors for classification. Definition adapted from [3,9,17,22,24].

Behavior	Description
Walking	The head moves forward/backward or sideways for at least two consecutive steps. From one place to another, the legs on the diagonal of the sheep move at the same time. Slow movement during grazing is excluded.
Standing	Sheep in standing position. The limbs and head are still or slightly moved, including standing chewing and ruminating.
Grazing	Sheep graze with their heads down, chew and move slowly to find grass.
Lying	Sheep in lying position. The head is down or up, and still or slightly moving. Chewing and ruminating are included.
Running	Sheep run faster to escape obstacles or catch up with other companions. In most cases, two front/rear legs move at the same time, and there is no biting or chewing.

**Table 3:** The total time and data size of observations available for each behavior. A row comprises an *x*-, *y*- and *z*-axis accelerometer and gyroscope raw data.

Behavior Number	Behavior	Total Time (s)	Total Data Rows
1	Walking	4352	87,040
2	Standing	15,499	309,980
3	Grazing	24,374	487,480
4	Lying	9534	190,680
5	Running	97	1940

**Table 4:** Features calculated for each time window (3, 5 and 11 s) based on *x*-, *y*- and *z*-axis accelerometer and gyroscope data. Equations adapted from [3,8,9,17,22,23].

Feature	The Number of Features	Equations/Description ^#^
Minimum value	6	Minimum value of all window values
Maximum value	6	Maximum value of all window values
Median	6	Median value of all window values
Upper quartile	6	Upper quartile value of all window values
Lower quartile	6	Lower quartile value of all window values
Kurtosis	6	Kurtosis calculated from window values
Skewness	6	Skewness calculated from window values
Range	6	Max−Min
Mean	6	$\frac{1}{n}\sum_{i=1}^{n}X\left(i\right)$
Variance	6	$\frac{1}{n-1}\sum_{i=1}^{n}{\left(X_{i}-\bar{X}\right)}^{2}$
Standard deviation	6	$\sqrt{\frac{1}{n-1}\sum_{i=1}^{n}{\left(X_{i}-\bar{X}\right)}^{2}}$
Root mean square (RMS)	6	$\sqrt{\frac{1}{n}\sum_{i=1}^{n}X_{i}{}^{2}}$
Signal magnitude area (SMA)	2	$\frac{1}{n}\sum_{i=1}^{n}\left(\left|X_{i}\right|+\left|Y_{i}\right|+\left|Z_{i}\right|\right)$
Energy	2	$\frac{1}{n}\sum_{i=1}^{n}{\left(X_{i}{}^{2}+Y_{i}{}^{2}+Z_{i}{}^{2}\right)}^{2}$
Entropy	2	$\frac{1}{n}\sum_{i=1}^{n}\left(1+{\left(X_{i}+Y_{i}+Z_{i}\right)}^{2}\right)\ln\left(1+{\left(X_{i}+Y_{i}+Z_{i}\right)}^{2}\right)$
Dominant frequency	6	After applying Fourier transformation, this is the frequency at which the signal has its highest power
Spectral energy	6	$\frac{1}{N}\sum_{k=1}^{N}{\left(F\left(k\right)\right)}^{2}$ $;N=\frac{n*f}{2}$
Spectral entropy	6	$-1\times \sum_{k=1}^{N}(\frac{F\left(k\right)}{Z_{1}N}\log_{2}\frac{F\left(k\right)}{Z_{1}N})$ $;Z_{1}=\frac{1}{N}\sum_{k=1}^{N}F\left(k\right)$
Vectorial dynamic body acceleration (VeDBA)	15	$\sqrt{a_{x}{}^{2}+a_{y}{}^{2}+a_{z}{}^{2}}$
Features of VeDBA		Minimum value, maximum value, median, upper quartile, lower quartile, kurtosis, skewness, range, mean, variance, standard deviation, RMS, dominant frequency, spectral energy, spectral entropy

^#^ Where *n* is the total number of all window values; where *N* is $\frac{n*f}{2}$, f is data collection frequency of the device (20 Hz).

**Table 5:** Summary of accuracy and kappa values for different ML predictions of walking, standing, grazing, lying and running at 3, 5 and 11 s time windows on the test dataset. Bold indicates the highest accuracy and Kappa values combination.

Time Window	Sensor	ELM		AdaBoost		Stacking		Number of Classified Behaviors
		Accuracy (%)	Kappa Value	Accuracy (%)	Kappa Value	Accuracy (%)	Kappa Value	
3 s	Accelerometer	91.5	0.871	94.7	0.921	94.6	0.918	5
	Gyroscope	90.1	0.850	92.4	0.885	93.1	0.895	5
	Accelerometer and Gyroscope	92.7	0.890	97.1	0.956	**97.2**	**0.959**	5
5 s	Accelerometer	93.3	0.899	97.5	0.963	97.6	0.964	4
	Gyroscope	93.0	0.894	95.0	0.923	94.9	0.922	4
	Accelerometer and Gyroscope	94.7	0.920	**98.9**	**0.983**	**98.9**	**0.983**	4
11 s	Accelerometer	98.26	0.983	99.3	0.989	99.3	0.988	4
	Gyroscope	97.0	0.951	96.6	0.945	96.2	0.939	4
	Accelerometer and Gyroscope	98.5	0.976	**99.7**	**0.995**	**99.7**	**0.995**	4

**Table 6:** Summary of the accuracy and Kappa values for different ML predictions of walking, standing, grazing, lying and running at 3, 5 and 11 s time windows on the CBS test dataset. Bold indicates the highest accuracy and Kappa values combination.

Time Window	Sensor	ELM		AdaBoost		Stacking		Number of Classified Behaviors
		Accuracy (%)	Kappa Value	Accuracy (%)	Kappa Value	Accuracy (%)	Kappa Value	
3 s	Accelerometer	84.8	0.796	80.2	0.735	81.2	0.749	5
	Gyroscope	82.8	0.770	78.9	0.716	77.6	0.700	5
	Accelerometer and Gyroscope	85.2	0.801	85.3	0.803	**87.8**	**0.836**	5
5 s	Accelerometer	83.2	0.774	78.9	0.717	83.8	0.782	4
	Gyroscope	82.7	0.767	78.6	0.711	81.4	0.750	4
	Accelerometer and Gyroscope	**87.4**	**0.830**	86.2	0.814	86.2	0.815	4
11 s	Accelerometer	72.7	0.631	76.9	0.689	74.4	0.656	4
	Gyroscope	66.3	0.542	63.9	0.510	64.8	0.524	4
	Accelerometer and Gyroscope	**78.0**	**0.702**	67.8	0.565	71.5	0.616	4

**Table 7:** Performance statistics of three optimal ML models (with the highest classification accuracy at the 3, 5 and 11 s time window on the CBS test dataset) used for classifying walking, standing, grazing, lying and running on the CBS test dataset.

Time Window	Model	Performance	Walking	Standing	Grazing	Lying	Running
3 s	Stacking	Precision (%)	99.3	81.9	98.5	75.8	90.3
		Recall (%)	95.4	72.8	100.0	85.4	75.7
		F-score (%)	97.3	77.1	99.3	80.3	82.4
5 s	ELM	Precision (%)	99.6	73.2	95.4	89.9	--
		Recall (%)	88.6	91.9	100.0	69.3	--
		F-score (%)	93.8	81.4	97.6	78.3	--
11 s	ELM	Precision (%)	97.9	63.3	82.4	92.5	--
		Recall (%)	59.1	94.6	100.0	52.6	--
		F-score (%)	73.7	75.9	90.4	67.1	--

**Table 8:** Summary of accuracy and the Kappa values for three ML models (with the highest classification accuracy at the 3, 5 and 11 s time windows on the CBS test dataset, trained by 12 features from three-axis accelerometer and three-axis gyroscope data) at the 3, 5 and 11 s time windows on the test dataset and the CBS test dataset separately.

	Time Window	Model	Accuracy (%)	Kappa Value
Test dataset	3 s	Stacking	95.1	0.927
	5 s	ELM	94.4	0.915
	11 s	ELM	98.4	0.975
CBS test dataset	3 s	Stacking	79.0	0.719
	5 s	ELM	78.2	0.707
	11 s	ELM	63.3	0.501

**Table 9:** Percentage of behavior time of the three grazing sheep for 7.5 h on pasture with three different sward surface heights (SSH).

SSH	B-Sheep	P-Sheep	R-Sheep	Average Proportion	
Short	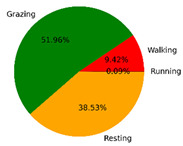	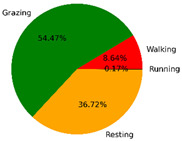	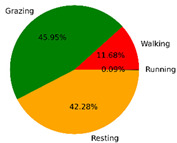	Walking	9.91%
				Grazing	50.79%
				Resting	39.18%
				Running	0.12%
Medium	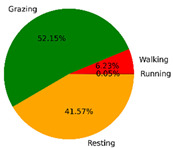	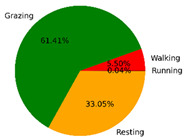	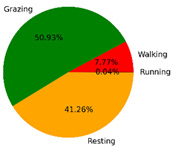	Walking	6.50%
				Grazing	54.83%
				Resting	38.63%
				Running	0.04%
Tall	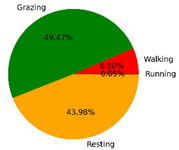	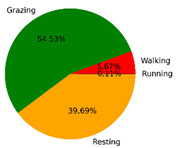	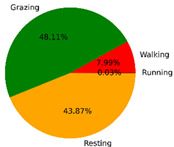	Walking	6.72%
				Grazing	50.70%
				Resting	42.52%
				Running	0.06%

## Data Availability

The data presented in this study are available upon request from the corresponding author.
